# Assessing the role of Chemokine (C–C motif) ligand 14 in AKI: a European consensus meeting

**DOI:** 10.1080/0886022X.2024.2345747

**Published:** 2024-04-26

**Authors:** Jay L. Koyner, Christian Arndt, Jaume Baldira Martinez de Irujo, Sílvia Coelho, Manuel Garcia-Montesinos de la Peña, Luca di Girolamo, Michael Joannidis, Pablo Jorge-Monjas, Christian Koch, Steven Lobaz, Alain Meyer, Marlies Ostermann, Nicoletta Pertica, John R. Prowle, Jon Silversides, Alexander Zarbock, Jorge Echeverri, Kai Harenski, Lui G. Forni

**Affiliations:** a Section of Nephrology, Department of Medicine, University of Chicago, Chicago, IL, USA; b Anesthesiology and Intensive Care Medicine, Philipps-Universitat Marburg Fachbereich Medizin, Marburg, Germany; c Department of Critical Care Medicine, Hospital Sant Pau, Barcelona, Spain; d Intensive Care Department, Hospital Fernando da Fonseca EPE, Amadora, Portugal; e Department of Intensive Care, Navarra University Hospital, Navarra, Spain; f Department of Anesthesia, Critical Care and Emergency, IRCCS Policlinico San Donato, Milan, Italy; g Division of Intensive Care and Emergency Medicine, Department of Internal Medicine, Medical Unversity Innsbruck, Innsbruck, Austria; h Anesthesiology and Critical Care Department, Clinic University Hospital of Valladolid, Valladolid, Spain; i Department of Anesthesiology, Operative Intensive Care Medicine and Pain Therapy, Justus Liebig University of Giessen, Giessen, Germany; j Anaesthetics and Intensive Care, Barnsley Hospital NHS Foundation Trust, Barnsley, UK; k Physiology and Functional Exploration Service, University Hospital of Strasbourg, Strasbourg, France; l Department of Critical Care & Nephrology, King’s College London, Guy’s & St Thomas’ Hospital, London, UK; m Division of Nephrology, Department of Biomedical and Surgical Sciences, University Hospital of Verona, Verona, Italy; n Critical Care & Perioperative Medicine Research Group, William Harvey Research Institute, Queen Mary University of London, London, UK; o Wellcome-Wolfson Institute for Experimental Medicine, Queen’s University, Belfast, UK; p Department of Anesthesiology, Intensive Care and Pain Medicine, University Hospital Münster, Münster, Germany; q Baxter Healthcare Corporation, Deerfield, IL, USA; r Baxter Deutschland GmbH, Unterschleissheim, Germany; s Critical Care Unit, Royal Surrey Hospital and School of Medicine, University of Surrey, Guildford, UK

**Keywords:** Biomarker testing experience, C‑C motif chemokine ligand 14 (CCL14), consensus, critical care nephrology, persistent acute kidney injury

## Abstract

**Background:**

Urinary Chemokine (C–C motif) ligand 14 (CCL14) is a biomarker associated with persistent severe acute kidney injury (AKI). There is limited data to support the implementation of this AKI biomarker to guide therapeutic actions.

**Methods:**

Sixteen AKI experts with clinical CCL14 experience participated in a Delphi-based method to reach consensus on when and how to potentially use CCL14. Consensus was defined as ≥ 80% agreement (participants answered with ‘Yes’, or three to four points on a five-point Likert Scale).

**Results:**

Key consensus areas for CCL14 test implementation were: identifying challenges and mitigations, developing a comprehensive protocol and pairing it with a treatment plan, and defining the target population. The majority agreed that CCL14 results can help to prioritize AKI management decisions. CCL14 levels above the high cutoff (> 13 ng/mL) significantly changed the level of concern for modifying the AKI treatment plan (*p* < 0.001). The highest level of concern to modify the treatment plan was for discussions on renal replacement therapy (RRT) initiation for CCL14 levels > 13 ng/mL. The level of concern for discussion on RRT initiation between High and Low, and between Medium and Low CCL14 levels, showed significant differences.

**Conclusion:**

Real world urinary CCL14 use appears to provide improved care options to patients at risk for persistent severe AKI. Experts believe there is a role for CCL14 in AKI management and it may potentially reduce AKI-disease burden. There is, however, an urgent need for evidence on treatment decisions and adjustments based on CCL14 results.

## Introduction

Acute kidney injury (AKI) is common amongst the critically ill with moderate to severe AKI, defined as KDIGO stage 2 or 3, occurring in approximately one third of individuals [1–4]. When severe AKI lasts 2 or more days, it is defined as persistent severe AKI (PS-AKI). PS-AKI may persist beyond 90 days, resulting in chronic kidney disease (CKD) [5,6]. Individuals with PS-AKI have an increased risk of morbidity and mortality [7–9] as well as a greater resource burden (longer length of hospital stay and greater post-discharge care costs) compared to those with transient severe AKI [10]. It follows that early identification of PS-AKI could facilitate evaluation and management strategies aimed at reducing the risk of further kidney damage and associated poor outcomes. However, use of serum creatinine and urine output, the current tools for defining and staging AKI [11], have limitations and cannot accurately or consistently predict the development of PS-AKI. Biomarkers that are reflective of kidney damage instead of kidney function could help stratify the risk of AKI persistence. While it has been proposed to include novel biomarkers in the definition of AKI, it has not been widely accepted [12].

Chemokine (C–C motif) ligand 14 (CCL14) is a urinary biomarker of persistent AKI that has been recently approved for clinical use in western Europe. Levels of CCL14 in urine predict AKI persistence in patients with moderate or severe AKI, and are not influenced by acute or chronic comorbidities [6]. In the RUBY study, persistent severe AKI was defined as Stage 3 AKI that lasted for more than 72 h [6]. High CCL14 levels have been associated with poor outcomes, including death and clinical indication for dialysis [6,13]. CCL14 cutoff values have been reported from the original RUBY study cohort (*n* = 335) to represent different levels of risk as follows [14]:

- CCL14 ≤ 1.3 ng/mL: Patient has the lowest risk of developing PS-AKI (*n =* 124, 37%)
- 1.3 ng/mL < CCL14 ≤ 13 ng/mL: Patient has increased risk of developing PS-AKI (*n =* 157, 47%)
- CCL14 > 13 ng/mL: Patient has the highest risk of developing PS-AKI (*n =* 54, 16%)

Currently, there is limited guidance to support the implementation of a biomarker to direct therapeutic actions in patients with moderate or severe AKI. The recent approval of CCL14 presents an opportunity for clinicians with experience using the biomarker to reach consensus on its role in patient care. Based on a modified Delphi method approach, an in-person round table meeting was convened. The goal of the meeting was to develop consensus recommendations to guide the future use of CCL14 in clinical practice and aid in AKI-care protocol development for new users.

## Material and methods

Participants included 16 intensivists and nephrologists from seven European countries who are all experts in AKI management and had approximately 12 months of experience using the CCL14 test. The session was chaired by a non-voting non-European moderator who also has expertise in AKI management and biomarker implementation (JLK).

**Figure 1.**
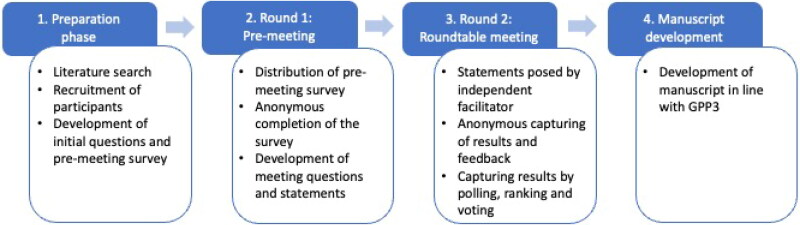
Overview of the four-step Delphi method used in the Roundtable Meeting. Each step was a distinct process that was completed before the following step was initiated. Results and discussions from each step were independently analysed and used to inform the direction and content of the following steps, e.g. if the group were split on a topic, then clarifying questions were crafted to guide the discussions in the following step(s) to identify and explore points of consensus or difference. GPP3, Good Publication Practice 3.

A modified Delphi-based method was used [15] to gather participants’ views and reach consensus whenever possible (Figure 1). A comprehensive pre-meeting survey was developed (by JE and JLK) to explore the use of CCL14 in clinical practice and potential topics for discussion. Participants responded to each question by a five-point Likert Scale (zero to four); a maximum score of 64 points could be reached for each question (4 points for 16 participants) (see for an example Supplemental Figure 1 and Table 1). The pre-meeting survey was followed by an in-person meeting, held in Munich, Germany, on 20 June 2023, which was attended live by 14 participants and virtually by two participants.

During the meeting, participants reviewed and discussed the results of the pre-meeting survey, provided input on five pre-determined areas of focus (biomarker implementation, target population, when to test, how to interpret test results, and potential therapeutic actions), and indicated their agreement (Yes or No), or their level of agreement on issues related to CCL14 use and testing using a five-point Likert Scale (zero to four).

Consensus was reached when ≥ 80% of the participants agreed (answered with ‘Yes’, or three to four points on the Likert Scale), and majority agreement if ≥ 50% of the participants agreed. No additional discussion or voting was conducted when consensus was not reached. An independent contract research organization (Avania B.V) was involved to ensure the anonymity of responses, data analysis, and manuscript writing.

## Post-Hoc statistical analysis

A post-hoc statistical analysis was performed on the level of concerns about modifying several areas of the AKI treatment plan between the CCL14 cutoff levels. Levels of concerns were described as mean (± standard deviation), and tables presenting the results of the post-hoc analysis show the mean difference with a 95% confidence interval. The post-hoc analysis include a comparison of both the mean and total assigned Likert Scale score of level of concern to modify the treatment plan between CCL14 cutoff levels. Additional details of the statistical analyses are described in the supplemental material.

## Results

### Background on CCL14 testing

The pre meeting survey revealed that test sampling was mostly done by a research team member (7/16, 43.8%), followed by nurses (6/16, 37.5%), or ICU physicians (5/16, 31.3%). Test analysis was usually done by research term members (6/16, 37.5%), central lab team members (6/16, 37.5%), or ICU physicians (4/16, 25%). In the majority of institutions, the CCL14 test was analyzed and centrifuged in the central lab (7/16, 43.8%), followed by the ICU lab/room (5/16, 31.3%) and a research lab/room (4/16, 25%).

### Requirements for implementing the CCL14 biomarker for AKI management

The pre-meeting survey revealed that the greatest challenges for CCL14 biomarker adoption were: lack of guidance and recommendations for patient management based on CCL14 results (49/64 points); and lack of sufficient evidence for specific clinical actions based on biomarker results (47/64 points) (Table 1). The median number of points and IQR can be found in Supplementary Table 1.

**Table 1.** Ranking of the challenges for adopting the chemokine (C-C motif) ligand 14 biomarker.

Challenges for adopting the CCL14 biomarker	Points (Max 64)
Lack of guidelines and recommendations for patient management based on CCL14 biomarker results	49
Insufficient evidence for specific clinical actions based on biomarker results	47
Lack of awareness of the role of the CCL14 biomarker	42
Internal cost containment policies	40
Lack of staffing resources	33
Lack of dedicated hospital educational resources	32
Laboratory department considerations	32
Administrative barriers	31
Other factors influencing clinicians’ decision-making	30
Lack of awareness of increased costs/economic burden associated with PS-AKI	29
Lack of awareness of persistent severe AKI (PS-AKI) and its impact on outcomes	27
Lack of routine AKI staging	26
Lack of specific local AKI management protocols	24
Low incidence of AKI Stage 2–3	12

The points assigned per challenge were: Not challenging = 0 points, Slightly Challenging = 1 point, Moderately challenging = 2 points, Very Challenging = 3 points, Extremely Challenging = 4 points.

In-meeting voting indicated consensus on the importance of the development of a comprehensive clinical protocol (87.5% agreement) (Supplementary Table 4), including different aspects such as personnel roles and responsibilities, target population criteria, interpretation of CCL14 test results according to cutoff values, with potential clinical interventions or changes in patient care (93.8% agreement) (Supplementary Table 5).

### Target patients for CCL14 testing

Populations deemed to most likely benefit from CCL14 testing were patients with AKI Stage 2 or 3 following cardiac surgery (53/64 points), and AKI stage 2 or 3 patients with heart failure (53/64 points), followed by patients with sepsis (52/64), or post-surgery (50/64 points), and any ICU patients with AKI stage 3 (50/64 points) (Table 2). The median number of points and IQR can be found in Supplementary Table 6.

**Table 2.** Ranking of populations likely to gain clinical benefit from chemokine (C-C motif) ligand 14 implementation.

Population	Points (Max 64)
AKI Stage 2 or 3 patients with cardiac surgery-associated AKI (CSA-AKI)	53
AKI Stage 2 or 3 patients with heart failure	53
AKI Stage 2 or 3 patients with sepsis	52
AKI Stage 2 or 3 surgery-associated AKI (other than cardiac) patients	50
All AKI Stage 3 patients	50
AKI Stage 2 or 3 patients with trauma	49
AKI Stage 2 or 3 in patients with risk factors for CKD (proteinuria, diabetes, etc.)	49
All AKI Stage 2 patients	47
AKI Stage 2 or 3 in patients with CKD	45

The points assigned per population were: Very Unlikely = 0 points, Somewhat Unlikely = 1 point, Equally Likely/Unlikely = 2 points, Somewhat Likely = 3 points, Very Likely = 4 points.

In-meeting voting resulted in unanimous consensus on the importance of defining the target population and creating an efficient strategy for AKI staging and target population identification prior to CCL14 implementation (Supplementary Table 8). The majority of sites are opting for once a day screening and very few sites use electronic alerting or nursing support to identify subjects (Supplementary Table 9).

### Timing of CCL14 testing and retesting

The participants agreed that several factors can influence the time window in which to test for CCL14, and that patients with stage 2 or 3 AKI who are candidates for CCL14 testing should be timely screened after clinical evaluation justifies assessment of risk for PS-AKI (Supplementary Table 10). CCL14 was considered most useful in cases of misalignment between a patient’s clinical condition and CCL14 results (Supplementary Table 11).

In the pre-meeting survey, re-testing was a higher priority in patients with initial CCL14 values indicating increased risk (45/64 points) and in patients with clinical instability (35/64 points) (Supplementary Table 12). There was 100% agreement or somewhat agreement that patients with intermediate CCL14 test values (> 1.30 ng/mL and ≤ 13 ng/mL) are potential candidates for re-testing, particularly in scenarios of clinical instability (Supplementary Table 13).

### Interpretation of CCL14 test results

Pre-meeting survey results indicated that CCL14 levels above the high cutoff (> 13 ng/mL) were considered the most helpful for adjustments in AKI management (53/64 points), followed by CCL14 levels below the low cutoff (≤ 1.3 ng/mL) (46/64 points) (Supplementary Table 14).

Both high and low CCL14 values were voted most likely to be clinically useful, with more than 80% of participants voting that they agreed or somewhat agreed with CCL14 utility in AKI management adjustment (Supplementary Table 15). Meeting voting also showed general agreement that CCL14 values indicating low risk (≤ 1.30 ng/mL) could help with de-escalation or maintenance of AKI management strategies (agreed or somewhat agreed by 87.5% of the participants). For CCL14 values > 13 ng/mL, all participants agreed or somewhat agreed that these levels can help identify patients at the highest risk of developing PS-AKI who could be candidates for increased prioritization of AKI management and care processes. Finally, 87.5% agreed or somewhat agreed that high CCL14 values could be useful in escalating AKI management, the level of care, and allocation of resources (Supplementary Table 15).

### Actions based on CCL14 test results

From the pre-meeting survey, CCL14 test results were considered the most helpful for decision-making on RRT initiation (55/64 points), and the least for decisions about the type of solution for fluid management (25/64 points) (Supplementary Table 16).

Table 3 includes rankings from the pre-meeting survey of the level of concern for modifying the treatment plan according to CCL14 levels. The level of concern for modifying the treatment plan was the highest for CCL14 levels above the high cutoff, and the lowest for CCL14 levels below the low cutoff. The level of concern was the highest for discussions on RRT initiation when CCL14 levels were above the high cutoff (48/64 points). The median number of points and IQR can be found in Supplementary Table 17.

**Table 3.** Ranking of the level of concern about modifying several areas of the treatment plan.

	Points (Max 64)		
Treatment Plan Area	CCL14 ≤ 1.30 ng/mL	CCL14 > 1.30 ng/mL and ≤ 13 ng/mL	CCL14 > 13 ng/mL
Discussions on renal replacement therapy initiation	16	44	48
Discussions with family on patient prognosis	23	37	42
Diuretic management	21	39	44
Drug dosing	23	35	41
Enhance diagnostic work-up (e.g. labs, images)	18	39	44
Exposure to nephrotoxins	23	39	44
Fluid management (type of solution)	17	28	36
Fluid management (volume)	19	37	46
Follow-up medical planning after discharge (outpatient care)	18	36	40
Hemodynamic management/monitoring	18	40	46
Nephrology consultation	14	31	35
Nutritional management	17	30	31
Strict urine output monitoring (e.g. foley catheter, continuous urinary output monitoring)	17	38	45

The points assigned per treatment area were: Not At All Concerned = 0 points, Slightly Concerned = 1 point, Moderately Concerned = 2 points, Very Concerned = 3 points, Extremely Concerned = 4 points.

The in-meeting voting results (Table 4) revealed general agreement that CCL14 test results can help support a patient-centred approach for timely and targeted clinical decisions in AKI management (agreed or somewhat agreed by 87.5%). The majority agreed that CCL14 results can help prioritize AKI management decisions, including decisions on fluid management (volume), diuretic use, strict urine output monitoring, nephrotoxic exposure, and hemodynamic management and monitoring. There was consensus that high risk CCL14 values can help prioritize discussions on RRT initiation while lower values can help avoid unnecessary RRT, and that overall CCL14 results can help prioritize AKI resources, care processes, and workflows.

**Table 4.** Consensus voting: Actions based on chemokine (C-C motif) ligand 14 biomarker results.

Consensus Statement	Response (*N* = 16)				
	Disagree *n* (%)	Somewhat disagree *n* (%)	Neither agree nor disagree *n* (%)	Somewhat agree *n* (%)	Agree *n* (%)
CCL14 biomarker results can help support a patient-centred approach for timely and targeted clinical decisions in patients with moderate or severe AKI when AKI management is the primary concern.	0 (0)	0 (0)	2 (12.5)	9 (56.3)	5 (31.3)
CCL14 biomarker results can help prioritize AKI resources, care processes, and workflows, including diagnostic work-up, monitoring, discussions with family on patient prognosis and follow-up planning.	0 (0)	1 (6.3)	2 (12.5)	5 (31.3)	8 (50.0)
CCL14 biomarker results can help prioritize AKI management decisions including fluid management (volume), diuretic use, strict urine output monitoring, and nephrotoxic exposure.	0 (0)	2 (12.5)	3 (18.8)	8 (50)	3 (18.8)
CCL14 biomarker results can help prioritize AKI management decisions, including hemodynamic management and monitoring.	0 (0)	3 (18.8)	4 (25)	7 (43.8)	2 (12.5)
CCL14 biomarker results can help prioritize discussions on RRT initiation in highest risk PS-AKI patients, and RRT avoidance in patients likely to recover renal function.	0 (0)	0 (0)	1 (6.3)	5 (31.3)	10 (62.3)
CCL14 biomarker results ≤ 1.30 ng/mL can be informative for certain aspects of AKI management and care processes of care, including drug dosing, exposure to nephrotoxins and family discussions on patient prognosis.	0 (0)	2 (12.5)	5 (31.3)	2 (12.5)	7 (43.8)
CCL14 biomarker results > 1.30 ng/mL and ≤ 13 ng/mL increase the level of concern to modify the AKI treatment plan, specific adjustment needs to be contextualized to the different clinical scenarios.	0 (0)	0 (0)	2 (12.5)	7 (43.8)	7 (43.8)
CCL14 biomarker results > 13 ng/mL increase the level of concern to modify the AKI treatment plan and are informative for certain aspects of AKI management and care processes, including fluid/hemodynamic management and monitoring.	0 (0)	1 (6.3)	3 (18.8)	5 (31.3)	7 (43.8)
CCL14 biomarker results > 13 ng/mL increase the level of concern to modify the AKI treatment plan and are informative for certain aspects of AKI management and care processes, including discussion on RRT initiation, strict urine output monitoring and diuretic management.	0 (0)	0 (0)	1 (6.3)	7 (43.8)	8 (50.0)

Majority agreement was reached for the statement that CCL14 values ≤ 1.30 ng/mL can be informative for certain aspects of AKI management and care processes, including drug dosing, exposure to nephrotoxins and family discussions on patient prognosis. For intermediate CCL14 values, 87.5% of participants agreed or somewhat agreed these values could increase the level of concern to modify the AKI treatment plan, when adjustments are contextualized to different clinical scenarios. The statement that high CCL14 values (> 13 ng/mL) could increase the level of concern to modify the AKI treatment plan and are informative for certain aspects of AKI management and care processes reached majority agreement when fluid/hemodynamic management and monitoring were included, but reached consensus when including RRT initiation, strict urine output monitoring and diuretic management.

### Post-Hoc analysis

A post-hoc analysis was performed on the difference between the CCL14 cutoff values and the level of concern for modifying the treatment plan. The means and standard deviations of the total score and Likert Scale level per CCL14 group are described in the supplement. Table 5 gives the mean difference between each possible pair of groups and presents the mean difference between each possible pair of groups comparing against one point difference of Likert Scale, assuming it as minimal importance difference. The data suggests a clinical difference in mean level of concern to modify the treatment plan between the high and low cutoff level of CCL14.

**Table 5.** Post-hoc analyses between chemokine (C-C motif) ligand 14cutoff levels.

	CCL14 Medium – Low		CCL14 High – Medium		CCL14 High – Low	
Points (max 64)						
	Mean difference (95% CI)	*p*-Value	Mean Difference (95% CI)	*p*-Value	Mean Difference (95% CI)	*p*-Value
Overall	17.6 (14.6, 20.7)	<0.001	5.3 (1.5, 9.1)	0.004	22.9 (19.6, 26.2)	<0.001
On Likert scale (0–4)						
Overall	1.1 (0.88, 1.32)	0.183	0.3 (0.11, 0.56)	>0.999	1.4 (1.19, 1.68)	<0.001

The mean difference test for level of concern to modify the treatment plan between the different levels of CCL14 was repeated for each treatment plan, see Table 6. Discussion on RRT initiation between High and Low and between Medium and Low level of CCL14 showed significant differences of respectively *p* = 0.023 and *p* = 0.009. In addition, hemodynamic management/monitoring, strict urine output monitoring, enhanced diagnostic work-up, and fluid management (volume) resulted in a trend toward significance (*p* < 0.1) for difference between High and Low level of CCL14. These outcomes show that changes in the test results impact the level of concern to modify various aspects of the treatment plan.

**Table 6.** Post-hoc analyses between chemokine (C-C motif) ligand 14cutoff values per treatment plan on likert scale.

	CCL14 Medium – Low		CCL14 High – Medium		CCL14 High – Low	
Treatment Plan Area	Mean Difference (95% CI)	*p*-Value	Mean Difference (95% CI)	*p*-Value	Mean Difference (95% CI)	*p*-Value
Discussions on renal replacement therapy (RRT) initiation	1.8 (1.02, 2.48)	0.023	0.3 (−0.46, 0.96)	0.980	2.0 (1.19, 2.81)	0.009
Discussions with family on patient prognosis	0.9 (0.07, 1.68)	0.623	0.3 (−0.47, 1.09)	0.959	1.2 (0.30, 2.08)	0.335
Diuretic management	1.1 (0.36, 1.89)	0.370	0.3 (−0.42, 1.05)	0.967	1.4 (0.60, 2.28)	0.147
Drug dosing	0.8 (−0.11, 1.61)	0.722	0.4 (−0.48, 1.23)	0.926	1.1 (0.21, 2.04)	0.391
Enhance diagnostic work-up (e.g. labs, images)	1.3 (0.62, 2.01)	0.182	0.3 (−0.42, 1.05)	0.967	1.6 (0.80, 2.45)	0.065
Exposure to nephrotoxins	1.0 (0.11, 1.89)	0.500	0.3 (−0.63, 1.25)	0.927	1.3 (0.28, 2.34)	0.270
Fluid management (type of solution)	0.7 (−0.23, 1.60)	0.755	0.5 (−0.50, 1.50)	0.841	1.2 (0.17, 2.21)	0.355
Fluid management (volume)	1.1 (0.27, 1.98)	0.383	0.6 (−0.32, 1.45)	0.839	1.7 (0.74, 2.64)	0.075
Follow-up medical planning after discharge (outpatient care)	1.1 (0.35, 1.90)	0.372	0.3 (−0.57, 1.07)	0.964	1.4 (0.50, 2.25)	0.193
Hemodynamic management/monitoring	1.4 (0.59, 2.16)	0.170	0.4 (−0.37, 1.12)	0.952	1.8 (0.88, 2.62)	0.044
Nephrology consultation	1.1 (0.18, 1.94)	0.443	0.3 (−0.66, 1.16)	0.949	1.3 (0.34, 2.28)	0.258
Nutritional management	0.8 (−0.07, 1.69)	0.666	0.1 (−0.84, 0.96)	0.979	0.9 (−0.11, 1.86)	0.601
Strict urine output monitoring (e.g. foley catheter, continuous urinary output monitoring)	1.3 (0.41, 2.21)	0.242	0.4 (−0.46, 1.33)	0.895	1.8 (0.82, 2.68)	0.054

## Discussion

We conducted a modified Delphi method round table expert panel on the use of CCL14 in the management of patients with stage 2/3 AKI, and identified several areas of interest and other areas of expert consensus. Protective interventions for patients with severe AKI can reduce AKI severity and its duration [16]. However, adherence to best practice measures is poor [17,18], in part due to insufficient prognostic information and the lack of adequate risk stratification tools [19], making a prognostic biomarker such as CCL14 essential. In areas with limited evidence, as is the case for AKI biomarker implementation and actions to be taken based on CCL14 results, a Delphi-based method offered insights from experienced users. These insights can help new institutions implement the CCL14 test (or other biomarkers), and guide clinical practice decision-making. Key areas of consensus for CCL14 test implementation were: identifying challenges and mitigation actions, developing a comprehensive protocol and action plan, and defining the target population.

The role of CCL14 in the pathobiology of AKI reflects processes involved in the development and progression of renal damage and renal repair, particularly macrophage trafficking and subsequent fibrosis [6]. CCL14 could therefore aid in prioritizing patients requiring closer monitoring, which was in line with the here obtained consensus for the helpfulness of CCL14 test results in the diagnostic work-up, monitoring, discussions with family on patient prognosis, and follow-up planning. The association of CCL14 with PS-AKI and non-recovery distinguishes this AKI biomarker from others, including Kidney Injury Molecule-1 and neutrophil gelatinase-associated lipocalin, which are early markers of kidney injury [20]. Moreover, changes in CCL14 level over time indicate a corresponding change in risk level for PS-AKI, meaning that serial CCL14 measurements may modify the assessment of risk over time [19].

Biomarkers able to distinguish between patients with PS-AKI and those with more transient disease will aid in patient management and perhaps the predictive enrichment in future AKI trials [21]. In our meeting, consensus was reached on the helpfulness of CCL14 results in prioritizing discussions on RRT initiation in those at the highest risk of developing PS-AKI, and avoidance of unnecessary RRT in patients likely to recover renal function. Meersch and colleagues were able to demonstrate in patients with established severe AKI, CCL14 levels were able to accurately identify which patients would develop a clinical indication for RRT [22]. Besides improved clinical outcomes, a patient-centred approach will likely result in reduction of healthcare costs. For example, the requirement for dialysis is a significant driver of the increased costs among patients with postoperative AKI [10], meaning that avoidance of unnecessary RRT may result in substantial cost reductions.

Data from the literature suggests that CCL14 could be supportive in other aspects of clinical decision-making as well, such as diuretic management. A significant interaction over time between urine output, CCL14 level, and diuretic use has been described, and a response to diuretics in patients with low CCL14 levels (<1.3 ng/mL) has only been recently reported [23]. If this data are further validated, it is anticipated that more clinicians would change their approach regarding fluid therapy based on CCL14 test results [24]. Interestingly, in our meeting, consensus was reached predominantly on the usefulness of CCL14 for RRT initiation discussions rather than on other areas of AKI management, which was verified by the post-hoc analysis. The heterogeneity of patients and interventions in the AKI care bundle and the lack of interventional clinical trials using the CCL14 test could explain this observation. Furthermore, a patient’s clinical condition was deemed important to consider, and the experts noted that some of the clinical interventions in AKI, such as hemodynamic or volume management, could generally be implemented before there is an indication for CCL14 testing. More evidence for actions to be taken based on CCL14 results is warranted. The lack of guidance and recommendations for patient management based on CCL14 results, and insufficient evidence for specific clinical actions based on biomarker results, were considered the biggest challenges for CCL14 biomarker adoption.

Our study has some limitations. First, the results shown here represent the points of view of AKI experts. In addition, participants were experienced users of the CCL14 test, and several were principal investigators in active clinical trials involving CCL14. The experiences of the participants may therefore differ from those of clinicians from non research-associated hospitals. Second, a relatively small number of clinicians from centers across Europe were included in the consensus meeting. Inputs from a larger group of clinicians, preferably from centers across the world, are required to support the findings and to apply the data to multiple regions world-wide. However, the CCL14 test is available only in Europe at the present time. Next, the post-hoc analysis performed here were for an exploratory purpose. Larger studies may be required to provide sufficient power.

Another limitation is that our study did not compare CCL14 with other biomarkers, such as NGAL and IL-18, for their use in clinical practice. Lastly, no specific therapy for severe AKI exists and there is currently no data supporting treatment decisions and adjustments based on CCL14 test results. Both issues are areas of intense research interest. A study investigating whether the implementation of a supportive extended care bundle in patients with a high-risk to develop PS-AKI (stratified by low and high CCL14 levels) can reduce the occurrence of persistent surgical AKI is currently ongoing [25].

In summary, the results outlined here indicate that CCL14 biomarker implementation requires development of a comprehensive protocol and action plan, definition of the target population, and creation of an efficient strategy to identify this population. CCL14 values above the high cutoff were considered the most helpful in clinical decision-making, especially for discussions on RRT initiation, strict urine output monitoring, and diuretic management. There is, however, an urgent need for evidence to support treatment decisions and treatment adjustment based on CCL14 results.

## Supplementary Material

Supplemental Material

## Data Availability

The data underlying this article are available in the article and in its online supplementary material.
