# Supplementary material for: Assessing the role of Chemokine (C–C motif) ligand 14 in AKI: a European consensus meeting
Source: Ren Fail. 2024 Apr 26;46(1):2345747. doi: 10.1080/0886022X.2024.2345747 (PMC11057457; doi:10.1080/0886022X.2024.2345747)
Supplement: Supplemental Material [file IRNF_A_2345747_SM5298.docx]

# Supplementary Methods

## Premeeting Survey

**Background**

1. Please provide details of the processes followed at your institution for use the CCL14 biomarker:
   1. Who is/are responsible for patient identification? (Multiple Answers)
      1. ICU physician
      2. Non-ICU physician
      3. Nurse
      4. Research team member
      5. Other (Open Answer)
   2. Who is/are responsible for test prescriptions? (Multiple Answers)
      1. ICU physician
      2. Non-ICU physician
      3. Nurse
      4. Research team member
      5. Other (Open Answer)
   3. Who is/are responsible for the test sampling? (Multiple Answers)
      1. ICU physician
      2. Non-ICU physician
      3. Nurse
      4. Research team member
      5. Other (Open Answer)
   4. Who is/are responsible for test sample centrifuge? (Multiple Answer)
2. Central lab team member
3. ICU physician
4. Non-ICU physician
5. Nurse
6. Research team member
7. Other (Open Answer)
   1. Where is the test sample centrifuge ? (Multiple Answer)
8. Central lab
9. ICU lab/room
10. Satellite lab
11. Research lab/room
12. Other (Open Answer)
    1. Who is/are responsible for test sample analysis (e.g., pipette sample and meter analysis)? (Multiple Answer)
13. Central lab team member
14. ICU physician
15. Non-ICU physician
16. Nurse
17. Research team member
18. Other (Open Answer)
    1. Where is the test sample analyzed (e.g., pipette sample and meter analysis)? (Single Answer)
19. Central lab
20. ICU lab
21. Satellite lab
22. Research lab
23. Other (Open Answer)
    1. How are the biomarker results documented? (Single Answer)
24. In the electronic medical record by a central lab team member
25. In the electronic medical record by a research team member
26. In the electronic medical record by an ICU team member
27. Manually registered in the medical history
28. Other (Open Answer)
    1. How are the results interpreted and how is the action plan defined? (Single Answer)
29. By guidance document/order sets linked in the EMR
30. By a guidance document posted in the ICU
31. By ICU physician discretion
32. By non-ICU physician discretion
33. Other (Open Answer)

**Workshop 1: Requirements to implement the CCL14 biomarker for AKI management**

1. In your opinion, before implementing the CCL14 biomarker in an institution:
   1. What hospital processes and key stakeholders may need to be considered or involved? (Open Answer)
   2. What type of preparatory activities and best practices are essential prior to implementation? (Open Answer)
   3. What are the minimum acceptable requirements to ensure good implementation of the CCL14 biomarker in clinical practice? (Open Answer)
   4. What are the most important benefits of implementing the CCL14 biomarker in an institution? (Open Answer)
2. How would you rate the following challenges for adopting the CCL14 biomarker in your institution? (Single answer for each challenge: 1. Not challenging, 2. Slightly challenging 3. Moderately challenging 4. Very challenging 5. Extremely challenging)
   1. Lack of awareness of persistent severe AKI (PS-AKI) and its impact on outcomes
   2. Lack of awareness of increased costs/economic burden associated with PS-AKI
   3. Lack of awareness of the role of the CCL14 biomarker
   4. Lack of routine AKI staging
   5. Low incidence of AKI Stage 2-3
   6. Insufficient evidence for specific clinical actions based on biomarker results
   7. Lack of specific local AKI management protocols
   8. Lack of guidelines and recommendations for patient management based on CCL14 biomarker results
   9. Other factors influencing clinicians’ decision-making
   10. Lack of dedicated hospital educational resources
   11. Lack of staffing resources
   12. Internal cost containment policies
   13. Laboratory department considerations
   14. Administrative barriers

3a. Have there been other challenges to adopting the CCL14 biomarker in your institution? (Open Answer)

1. How would you rate the importance of the following implementation action plans to ensure proper use of the CCL14 biomarker in clinical practice? (Single answer for each action plan: 1. Not Important, 2. Slightly important, 3. Moderately important, 4. Very Important, 5. Extremely important).
   1. Preparatory meetings with relevant clinical stakeholders (e.g., physician consultants and attendees, nurses)
   2. Preparatory meetings with other relevant healthcare stakeholders (e.g., laboratory personnel, pharmacists)
   3. Preparatory meetings with relevant non-clinical stakeholders (e.g., administrators, managers)
   4. Development of a clinical protocol introducing the CCL14 biomarker as part of the clinical workflow
   5. Ensuring protocol acceptance and dissemination among hospital stakeholders before the CCL14 biomarker is introduced in clinical practice
   6. Training in the technology to measure the CCL14 biomarker
   7. Education plans to increase disease condition awareness, patient identification, and protocol training
   8. Identification of metrics for CCL14 biomarker adoption, operational success, and clinical care impact
   9. Definition of good documentation practices for CCL14 biomarker results in reporting, protocol actions, and success metrics
   10. Periodic review meetings with clinical stakeholders to discuss progress and/or problems, clinical cases, educational needs, protocol updates, and adaptative plans

4a. Are any other implementation action plans needed to ensure the proper use of the CCL14 biomarker in clinical practice? (Open Answer)

1. How would you rate the importance of the following components of a CCL14 biomarker protocol? (Single answer for each protocol component: 1. Not Important, 2. Slightly important, 3. Moderately important, 4. Very Important, 5. Extremely important)
   1. Roles and responsibilities for protocol-involved personnel
   2. Identification of the target population criteria (who to test, who not to test)
   3. When and how to perform measurements of the CCL14 biomarker
   4. General recommendations for CCL14 biomarker workflow
   5. Interpretation of CCL14 biomarker results according to test cut-offs
   6. Bundle of clinical interventions/goals according to risk stratification
   7. Changes in patient care resources, logistics, and/or processes according to risk stratification
   8. Determination of the minimum information to document in the EMR

5a. What other components and details should a CCL14 biomarker protocol include that would facilitate test integration in the clinical workflow? **[If available, provide the synopsis of the protocol you used in your institution to analyze the common elements and rank during the live meeting]** (Open Answer)

**Workshop 2: Who are the target patients for the CCL14 biomarker?**

1. How likely are the following populations to gain clinical benefit from implementing the CCL14 biomarker? (Single answer for each population: 1. Very unlikely, 2. Somewhat unlikely, 3. Equally likely/unlikely, 4. Somewhat likely, 5. Very likely)
   1. All AKI Stage 2 patients
   2. All AKI Stage 3 patients
   3. AKI Stage 2 or 3 patients with sepsis
   4. AKI Stage 2 or 3 patients with heart failure
   5. AKI Stage 2 or 3 patients with cardiac surgery-associated AKI (CSA-AKI)
   6. AKI Stage 2 or 3 surgery-associated AKI (other than cardiac) patients
   7. AKI Stage 2 or 3 patients with trauma
   8. AKI Stage 2 or 3 in patients with CKD
   9. AKI Stage 2 or 3 in patients with risk factors for CKD (proteinuria, diabetes, etc.)

6a. In your opinion, what patients are appropriate candidates for the CCL14 biomarker? (Open Answer)

1. During your experience with the CCL14 biomarker, how often did you use the following strategies to facilitate patient screening and identification? (Single answer for each strategy: 1. Never, 2. Rarely, 3. Sometimes, 4 Often, 5. Always)
   1. Electronic/ automated AKI staging
   2. Once daily chart review by the physician provider
   3. Twice daily chart review by the physician provider
   4. More than twice daily chart review by the physician provider
   5. ICU nurse AKI staging with standard guidelines and manual chart checking
   6. External physician (e.g., research fellow) AKI staging with standard guidelines and manual chart checking

7a. What other strategies did you use to facilitate patient screening and identification during your experience with the CCL14 biomarker? (Open Answer)

**Workshop 3: When are patients being tested and re-tested?**

1. How would you rate the priority level to order a CCL14 biomarker test in the following situations? (Single answer for each moment: 1. Not a priority, 2. Low priority, 3. Medium priority, 4. High priority, 5. Essential)
   1. When a patient reaches Stage 2 AKI (defined by serum creatinine)
   2. When a patient reaches Stage 2 AKI (defined by urine output)
   3. When a patient reaches Stage 3 AKI (defined by serum creatinine)
   4. When a patient reaches Stage 3 AKI (defined by urine output)
   5. 24-48 hours after a patient reaches Stage 2 AKI with no response to clinical management
   6. 24-48 hours after a patient reaches Stage 3 AKI with no response to clinical management
   7. When a furosemide stress test is performed
   8. When a furosemide stress test is negative
   9. When considering RRT initiation as an alternative treatment
2. Is there a time window during which the CCL14 biomarker could provide the best information? (Yes/No, Explain)
3. How would you rate the priority level for additional/serial CCL14 testing in the following situations? (Single answer for each situation: 1. Not a priority, 2. Low priority, 3. Medium priority, 4. High priority, 5. Essential)
   1. A prior CCL14 value indicating highest risk for persistent severe AKI(e.g., > 13 ng/mL)
   2. A prior CCL14 value indicating increased risk for persistent severe AKI (e.g., > 1.30 ng/mL and ≤ 13 ng/mL )
   3. A prior low CCL14 value indicating lowest risk of developing persistent severe AKI (e.g., ≤ 1.30 ng/mL)
   4. Clinical instability
   5. Clinical improvement

10a. What other triggers would you consider for additional/serial CCL14 testing? (Open Answer)

1. How would you rate the frequency of assessment if a patient is a candidate for additional/serial CCL14 testing ? (Single answer for each frequency: 1. Never, 2. Rarely, 3. Sometimes, 4 Often, 5. Always)

a. 12 hours after the initial CCL14 results

b. 24 hours after the initial CCL14 results

c. 48 hours after the initial CCL14 results

d. Other, please specify (Open Answer)

**Workshop 4:** **How are quantitative CCL14 biomarker results being interpreted?**

1. What is your level of clinical concern (e.g., outcomes or AKI management adjustment) according to the following initial CCL14 biomarker results? (Single answer for each threshold: 1. Not at all concerned, 2. Slightly concerned, 3. Moderately concerned, 4. Very concerned, 5. Extremely concerned)
   1. CCL14 biomarker results ≤ 1.30 ng/mL
   2. CCL14 biomarker results > 1.30 ng/mL and ≤ 13 ng/mL
   3. CCL14 biomarker results > 13 ng/mL
2. How helpful would you rate the CCL14 biomarker results for AKI management adjustment according to the following thresholds? (Single answer for each threshold: 1. Definitely not helpful, 2. Likely not helpful, 3. Equally unhelpful/helpful, 4. Likely helpful, 5. Definitely helpful)
3. CCL14 biomarker results ≤ 1.30 ng/mL
4. CCL14 biomarker results > 1.30 ng/mL and ≤ 13 ng/mL
5. CCL14 biomarker results > 13 ng/mL
6. How helpful would you rate CCL14 biomarker results **≤ 1.30 ng/mL** (lowest risk) for the following bundle-care actions? (Single answer for each action: 1. Definitely not helpful, 2. Likely not helpful, 3. Equally unhelpful/helpful, 4. Likely helpful, 5. Definitely helpful)
7. De-escalate the AKI treatment (e.g., fluids, pressors, drugs) according to your AKI bundle of care
8. Maintain general measures for the AKI treatment
9. De-escalate the level of care and allocated resources (e.g., monitoring, diagnostics, staff, level of critical care) according to your AKI bundle
10. Maintain general measures for the level of care and allocated resources
11. How helpful would you rate CCL14 biomarker results **> 1.30 ng/mL and ≤ 13 ng/mL (increased risk)** for the following bundle of care actions? (Single answer for each action: 1. Definitely not helpful, 2. Likely not helpful, 3. Equally unhelpful/helpful, 4. Likely helpful, 5. Definitely helpful)
12. Escalate the AKI treatment (e.g., fluids, pressors, drugs) according to your AKI bundle of care
13. Maintain general measures for monitoring and managing AKI
14. Escalate the level of care and allocated resources (e.g., monitoring, diagnostics, staff, level of critical care) according to your AKI bundle of care.
15. Maintain general measures for the level of care and allocated resources
16. How helpful would you rate CCL14 biomarker results **> 13 ng/mL (highest risk)** for the following bundle of care actions? (Single answer for each action: 1. Definitely not helpful, 2. Likely not helpful, 3. Equally unhelpful/helpful, 4. Likely helpful, 5. Definitely helpful)
17. Escalate the AKI treatment (e.g., fluids, pressors, drugs) according to your AKI bundle of care
18. Maintain general measures for monitoring and managing AKI
19. Escalate the level of care and allocated resources (e.g., monitoring, diagnostics, staff) according to your AKI bundle of care
20. Maintain general measures for the level of care and allocated resources

**Workshop 5:** **What actions are taken based on CCL14 biomarker results?**

1. How helpful would the CCL14 biomarker results be for informing decisions on AKI management in the following areas: (Single answer for each area: 1. Definitely not helpful, 2. Likely not helpful, 3. Equally unhelpful/helpful, 4. Likely helpful, 5. Definitely helpful)
2. Fluid management (volume)
3. Fluid management (type of solution)
4. Hemodynamic management/monitoring
5. Diuretic management
6. Strict urine output monitoring (e.g., foley catheter, continuous urinary output monitoring)
7. Drug dosing
8. Exposure to nephrotoxins
9. Nutritional management
10. Nephrology consultation
11. Discussions on renal replacement therapy (RRT) initiation
12. Enhance diagnostic work-up (e.g., labs, images)
13. Discussions with family on patient prognosis
14. Follow-up medical planning after discharge (outpatient care)
15. What is your level of concern about modifying your treatment plan for the following areas when the CCL14 biomarker result is **≤ 1.30 ng/mL**? (Single answer for each threshold: 1. Not at all concerned, 2. Slightly concerned, 3. Moderately concerned, 4. Very concerned, 5. Extremely concerned)
16. Fluid management (volume)
17. Fluid management (type of solution)
18. Hemodynamic management/monitoring
19. Diuretic management
20. Strict urine output monitoring (e.g., foley catheter, continuous urinary output monitoring)
21. Drug dosing
22. Exposure to nephrotoxins
23. Nutritional management
24. Nephrology consultation
25. Discussions on renal replacement therapy (RRT) initiation
26. Enhance diagnostic work-up (e.g., labs, images)
27. Discussions with families on patient prognosis
28. Follow-up medical planning after discharge (outpatient care)
29. What is your level of concern about modifying your treatment plan for the following areas when the CCL14 biomarker result is **> 1.30 ng/mL and ≤ 13 ng/mL**? (Single answer for each threshold: 1. Not at all concerned, 2. Slightly concerned, 3. Moderately concerned, 4. Very concerned, 5. Extremely concerned)
30. Fluid management (volume)
31. Fluid management (type of solution)
32. Hemodynamic management/monitoring
33. Diuretic management
34. Strict urine output monitoring (e.g., foley catheter, continuous urinary output monitoring)
35. Drug dosing
36. Exposure to nephrotoxins
37. Nutritional management
38. Nephrology consultation
39. Discussions on renal replacement therapy (RRT) initiation
40. Enhance diagnostic work-up (e.g., labs, images)
41. Discussions with family on patient prognosis
42. Follow-up medical planning after discharge (outpatient care)
43. What is your level of concern about modifying your treatment plan for the following areas when the CCL14 biomarker result is **> 13 ng/mL**? (Single answer for each threshold: 1. Not at all concerned, 2. Slightly concerned, 3. Moderately concerned, 4. Very concerned, 5. Extremely concerned)
44. Fluid management (volume)
45. Fluid management (type of solution)
46. Hemodynamic management/monitoring
47. Diuretic management
48. Strict urine output monitoring (e.g., foley catheter, continuous urinary output monitoring)
49. Drug dosing
50. Exposure to nephrotoxins
51. Nutritional management
52. Nephrology consultation
53. Discussions on renal replacement therapy (RRT) initiation
54. Enhance diagnostic work-up (e.g., labs, images)
55. Discussions with family on patient prognosis
56. Follow-up medical planning after discharge (outpatient care)

## Statistical Analysis

A post-hoc statistical analysis was performed on the level of concerns about modifying several areas of the AKI treatment plan between the CCL14 cut-off levels. Summary of levels of concerns were described as mean (± standard deviation), and tables presenting the results of the Post-Hoc Analyses show the mean difference with a 95% confidence interval. The post-hoc analyses include a comparison of both the mean and total assigned Likert Scale score of level of concerns to modify the treatment plan between CCL14 cut-off levels.

T-tests were used. The test of the sum of the points is the one-sided superiority t-test with the significance level of 0.025 against the margin of 0 point. Since it is not straightforward to establish a meaningful superiority margin for the difference between total scores, a margin of 0 was used for these tests. However, to be able to gain interpretable results, a one-sided t-test with significance level of 0.025 was performed on the mean of the scores. Since every 1-point increment of 5-point Likert Scale has a different clinical interpretation, superiority margin of 1 point was used for the test of superiority for the means.

To gain better insight on the level of concern for modifying each treatment plan, the mean difference test on Likert Scale for level concern between the different levels of CCL14 was repeated for each treatment plan. The post-hoc analysis is performed for exploratory purposes; therefore no correction of alpha error was considered for these tests.

# Supplementary results

## Workshop 1: What are the requirements for implementing the CCL14 biomarker for AKI management?

### Pre-meeting survey questions and results

1. How would you rate the following challenges for adopting the CCL14 biomarker in your institution? (Single answer for each challenge: 1. Not challenging, 2. Slightly challenging 3. Moderately challenging 4. Very challenging 5. Extremely challenging)
   1. Lack of awareness of persistent severe AKI (PS-AKI) and its impact on outcomes
   2. Lack of awareness of increased costs/economic burden associated with PS-AKI
   3. Lack of awareness of the role of the CCL14 biomarker
   4. Lack of routine AKI staging
   5. Low incidence of AKI Stage 2-3
   6. Insufficient evidence for specific clinical actions based on biomarker results
   7. Lack of specific local AKI management protocols
   8. Lack of guidelines and recommendations for patient management based on CCL14 biomarker results
   9. Other factors influencing clinicians’ decision-making
   10. Lack of dedicated hospital educational resources
   11. Lack of staffing resources
   12. Internal cost containment policies
   13. Laboratory department considerations
   14. Administrative barriers

Results of question 1 are provided in **Figure 1**. **Table 1** presents an overview of the ranking of these results.

**Figure 1.** Challenges for adopting the CCL14 biomarker

**Table 1.** Ranking of the Challenges for Adopting the CCL14 Biomarker

| Challenges for adopting the CCL14 biomarker | Median (IQR) |
| --- | --- |
| Lack of guidelines and recommendations for patient management based on CCL14 biomarker results | 3 (3; 3.25) |
| Insufficient evidence for specific clinical actions based on biomarker results | 3 (2; 4) |
| Lack of awareness of the role of the CCL14 biomarker | 3 (2; 3) |
| Internal cost containment policies | 3 (1; 3.25) |
| Lack of staffing resources | 2 (1; 3) |
| Lack of dedicated hospital educational resources | 2 (1; 3) |
| Laboratory department considerations | 2 (1; 3) |
| Administrative barriers | 2 (1; 3) |
| Other factors influencing clinicians' decision-making | 2 (1; 2.25) |
| Lack of awareness of increased costs/economic burden associated with PS-AKI | 1.5 (1; 2.25) |
| Lack of awareness of persistent severe AKI (PS-AKI) and its impact on outcomes | 2 (0.75; 2.25) |
| Lack of routine AKI staging | 2 (1; 2) |
| Lack of specific local AKI management protocols | 1 (0.75; 2) |
| Low incidence of AKI Stage 2-3 | 0 (0; 1) |
| The points assigned per challenge were: Not challenging = 0 points, Slightly Challenging = 1 point, Moderately challenging = 2 points, Very Challenging = 3 points, Extremely Challenging = 4 points | |

1. How would you rate the importance of the following implementation action plans to ensure proper use of the CCL14 biomarker in clinical practice? (Single answer for each action plan: 1. Not Important, 2. Slightly important, 3. Moderately important, 4. Very Important, 5. Extremely important).
   1. Preparatory meetings with relevant clinical stakeholders (e.g., physician consultants and attendees, nurses)
   2. Preparatory meetings with other relevant healthcare stakeholders (e.g., laboratory personnel, pharmacists)
   3. Preparatory meetings with relevant non-clinical stakeholders (e.g., administrators, managers)
   4. Development of a clinical protocol introducing the CCL14 biomarker as part of the clinical workflow
   5. Ensuring protocol acceptance and dissemination among hospital stakeholders before the CCL14 biomarker is introduced in clinical practice
   6. Training in the technology to measure the CCL14 biomarker
   7. Education plans to increase disease condition awareness, patient identification, and protocol training
   8. Identification of metrics for CCL14 biomarker adoption, operational success, and clinical care impact
   9. Definition of good documentation practices for CCL14 biomarker results in reporting, protocol actions, and success metrics
   10. Periodic review meetings with clinical stakeholders to discuss progress and/or problems, clinical cases, educational needs, protocol updates, and adaptative plans

Results of question 2 are provided in **Figure 2**. **Table 2** presents an overview of the ranking of these results.

**Figure 2.** Importance of implementation of action plans

**Table 2.** Ranking of the implementation of action plans by importance

| **Action plan** | **Points**  **(Max 64)** |
| --- | --- |
| Preparatory meetings with relevant clinical stakeholders (e.g., physician consultants and attendees, nurses) | 57 |
| Development of a clinical protocol introducing the CCL14 biomarker as part of the clinical workflow | 52 |
| Identification of metrics for CCL14 biomarker adoption, operational success, and clinical care impact | 52 |
| Definition of good documentation practices for CCL14 biomarker results in reporting, protocol actions, and success metrics | 50 |
| Preparatory meetings with other relevant healthcare stakeholders (e.g., laboratory personnel, pharmacists) | 48 |
| Periodic review meetings with clinical stakeholders to discuss progress and/or problems, clinical cases, educational needs, protocol updates, and adaptative plans | 46 |
| Education plans to increase disease condition awareness, patient identification, and protocol training | 46 |
| Training in the technology to measure the CCL14 biomarker | 45 |
| Ensuring protocol acceptance and dissemination among hospital stakeholders before the CCL14 biomarker is introduced in clinical practice | 44 |
| Preparatory meetings with relevant non-clinical stakeholders (e.g., administrators, managers) | 38 |
| The points assigned per challenge were: Not Important = 0 points, Slightly Important = 1 point, Moderately Important = 2 points, Very Important = 3 points, Extremely Important = 4 points | |

1. How would you rate the importance of the following components of a CCL14 biomarker protocol? (Single answer for each protocol component: 1. Not Important, 2. Slightly important, 3. Moderately important, 4. Very Important, 5. Extremely important)
   1. Roles and responsibilities for protocol-involved personnel
   2. Identification of the target population criteria (who to test, who not to test)
   3. When and how to perform measurements of the CCL14 biomarker
   4. General recommendations for CCL14 biomarker workflow
   5. Interpretation of CCL14 biomarker results according to test cut-offs
   6. Bundle of clinical interventions/goals according to risk stratification
   7. Changes in patient care resources, logistics, and/or processes according to risk stratification
   8. Determination of the minimum information to document in the EMR

Results of question 3 are provided in **Figure 3**. **Table 3** presents an overview of the ranking of these results.

**Figure 3.** Importance of CCL14 protocol components

**Table 3.** Ranking of the CCL14 protocol components by importance

| **CCL14 biomarker protocol components** | **Points**  **(Max 64)** |
| --- | --- |
| Identification of the target population criteria (who to test, who not to test) | 58 |
| When and how to perform measurements of the CCL14 biomarker | 56 |
| Interpretation of CCL14 biomarker results according to test cut-offs | 55 |
| Bundle of clinical interventions/goals according to risk stratification | 52 |
| General recommendations for CCL14 biomarker workflow | 47 |
| Changes in patient care resources, logistics, and/or processes according to risk stratification | 42 |
| Roles and responsibilities for protocol-involved personnel | 41 |
| Determination of the minimum information to document in the EMR | 40 |
| The points assigned per challenge were: Not Important = 0 points, Slightly Important = 1 point, Moderately Important = 2 points, Very Important = 3 points, Extremely Important = 4 points | |

### Voting statements and results

**Table 4.** Yes/No consensus voting workshop 1

| Consensus Statement | Response (N=16) | |
| --- | --- | --- |
|  | **Yes**  **n (%)** | **No**  **n (%)** |
| Do you agree on the importance of identifying the challenges (e.g., lack of awareness, protocol-related, education/training, engagement of different stakeholders) and potential mitigation actions for adoption of the CCL14 biomarker at an institution? | 15 (93.8) | 1 (6.3) |
| Do you agree with the importance of developing an implementation action plan before using the CCL14 biomarker? (e.g., preparatory meetings, protocol development, training & education strategy, and follow-up metrics & activities)? | 15 (93.8) | 1 (6.3) |
| Do you agree with the importance of developing a comprehensive clinical protocol in an institution planning to use the CCL14 biomarker? | 14 (87.5) | 2 (12.5) |

**Table 5.** Level of agreement consensus voting workshop 1

| Consensus Statement | Response (N=16) | | | | |
| --- | --- | --- | --- | --- | --- |
|  | Disagree  n (%) | Somewhat Disagree  n (%) | Neither Agree nor Disagree  n (%) | Somewhat Agree  n (%) | Agree  n (%) |
| In preparation for introducing the CCL14 biomarker at an institution, clinicians should consider assessing potential challenges, such as lack of disease and biomarker awareness, protocol development and implementation challenges, insufficient education & training resources, and lack of engagement of different stakeholders. | 0 (0) | 0 (0) | 2 (12.5) | 1 (6.3) | 13 (81.3) |
| To properly use the CCL14 biomarker in clinical practice, clinicians need to consider different elements of an implementation action plan such as preparatory meetings with relevant clinical and non-clinical stakeholders, development and dissemination of the clinical protocol, education and training strategy, follow-up metrics & activities. | 0 | 1 (6.3) | 2 (12.5) | 2 (12.5) | 11 (68.8) |
| To enable proper use of the CCL14 biomarker, institutions need to consider developing a comprehensive clinical protocol, including different components such as personnel roles and responsibilities, target population criteria, interpretation of CCL14 biomarker results according to test cut-offs and potential clinical interventions, and changes in patient care, information to document. | 0 (0) | 1 (6.3) | 0 (0) | 4 (25) | 11 (68.8) |

## Workshop 2: Who are the target patients for the CCL14 biomarker?

### Voting statements and results

**Table 6**. Populations Likely to Gain Clinical Benefit from Chemokine (C-C motif) Ligand 14 Implementation

| Population | Median (IQR) |
| --- | --- |
| AKI Stage 2 or 3 patients with cardiac surgery-associated AKI (CSA-AKI) | 3.5 (3 ;4) |
| AKI Stage 2 or 3 patients with heart failure | 3.5 (3 ;4) |
| AKI Stage 2 or 3 patients with sepsis | 3 (3; 4) |
| AKI Stage 2 or 3 surgery-associated AKI (other than cardiac) patients | 3 (2.75; 4) |
| All AKI Stage 3 patients | 3 (2.75; 4) |
| AKI Stage 2 or 3 patients with trauma | 3 (2; 4) |
| AKI Stage 2 or 3 in patients with risk factors for CKD (proteinuria, diabetes, etc.) | 3 (3; 4) |
| All AKI Stage 2 patients | 3 (2.75; 4) |
| AKI Stage 2 or 3 in patients with CKD | 3 (2; 3.25) |
| The points assigned per population were: Very Unlikely = 0 points, Somewhat Unlikely = 1 point, Equally Likely/Unlikely = 2 points, Somewhat Likely = 3 points, Very Likely = 4 points. | |

**Table 7.** Level of agreement consensus voting workshop 2

| Consensus Statement | Response (N=16) | | | | |
| --- | --- | --- | --- | --- | --- |
|  | Disagree  n (%) | Somewhat Disagree  n (%) | Neither Agree nor Disagree  n (%) | Somewhat Agree  n (%) | Agree  n (%) |
| Patients with moderate to severe (stage 2 or 3) AKI are candidates for assessment of the risk for developing PS-AKI. | 0 (0) | 0 (0) | 0 (0) | 6 (37.5) | 10 (62.5) |
| The patient groups who would derive a significant clinical benefit from stratifying the risk of PS-AKI should be considered initial candidates for screening in institutions where the CCL14 biomarker is available. | 0 (0) | 1 (6.3) | 1 (6.3) | 7 (43.8) | 7 (43.8) |
| Patient groups who would derive a significant clinical benefit from stratifying PS-AKI risk include those with either AKI Stage 3, or AKI Stage 2 or 3 within specific scenarios (including CSA-AKI, heart failure, sepsis, surgery-associated AKI). | 0 (0) | 1 (6.3) | 2 (12.5) | 5 (31.3) | 8 (50.0) |

**Table 8.** Yes/No consensus voting workshop 2

| Consensus Statement | Response (N=16) | |
| --- | --- | --- |
|  | **Yes**  **n (%)** | **No**  **n (%)** |
| Do you agree on the importance of defining the target population for the CCL14 biomarker before an institution implements the biomarker in clinical practice? | 16 (100) | 0 (0) |
| Do you agree with the importance of creating an efficient strategy for AKI staging and target population identification before implementing the CCL14 biomarker in an institution? | 16 (100) | 0 (0) |

**Table 9.** Consensus voting workshop 2: Efficiency of patient detection and identification strategies

|  | **Response (N=16)** | | | | |
| --- | --- | --- | --- | --- | --- |
| **Strategy** | **Inefficient**  **n (%)** | **Somewhat Inefficient**  **n (%)** | **Neither Efficient nor Inefficient**  **n (%)** | **Somewhat Efficient**  **n (%)** | **Efficient**  **n (%)** |
| Electronic/ automated AKI staging | 4 (25.0) | 2 (12.5) | 3 (18.8) | 5 (31.2) | 7 (12.5) |
| ICU nurse AKI staging with standard guidelines and manual chart checking | 5 (31.2) | 5 (31.2) | 2 (12.5) | 2 (12.5) | 2 (12.5) |
| More than twice daily chart review by the physician provider | 6 (37.5) | 1 (6.3) | 4 (25) | 4 (25) | 1 (6.3) |
| Once daily chart review by the physician provider | 0 (0.0) | 2 (12.5) | 2 (12.5) | 5 (31.2) | 7 (43.8) |
| Twice daily chart review by the physician provider | 0 (0.0) | 2 (12.5) | 1 (6.3) | 8 (50.0) | 5 (31.8) |

## Workshop 3: When are patients being tested and retested?

### Part 1: Voting statements and results

**Table 10.** Level of agreement consensus voting workshop 3, Part 1

| Consensus Statement | Response (N=16) | | | | |
| --- | --- | --- | --- | --- | --- |
|  | **Disagree**  **n (%)** | **Somewhat Disagree**  **n (%)** | **Neither Agree nor Disagree**  **n (%)** | **Somewhat Agree**  **n (%)** | **Agree**  **n (%)** |
| Several factors (e.g., clinical scenarios, AKI severity, AKI staging practices, response to clinical treatment, and uncertainty about RRT) can influence the time window in which to screen for CCL14. | 0 (0) | 0 (0) | 1 (6.3) | 5 (31.2) | 10 (62.5) |
| Patients with stage 2 or 3 AKI who are candidates for CCL14 biomarker testing should be timely screened after clinical evaluation justifies risk assessment for developing PS-AKI. | 0 (0) | 0 (0) | 1 (6.3) | 9 (56.3) | 6 (37.5) |

Rank the following scenarios according to your level of interest to know the CCL14 values:

The results of the poll voting question are presented in **Table 10**.

**Table 11**. Ranking of the Scenarions According to the Level of Interest to Know CCL14 Values

| Scenario | | Rating of Clinical Interest (N=16) | | | |
| --- | --- | --- | --- | --- | --- |
| **CCL14 result** | **Clinical instabillity** | **1^st^**  **n (%)** | **2^nd^**  **n (%)** | **3^rd^**  **n (%)** | **4^th^**  **n (%)** |
| High | Low | 9 (56.3) | 1 (6.3) | 6 (37.5) | 0 (0) |
| Low | High | 4 (25.0) | 5 (31.3) | 1 (6.3) | 6 (37.5) |
| High | High | 3 (18.8) | 3 (18.8) | 7 (43.8) | 3 (18.8) |
| Low | Low | 0 (0) | 7 (43.8) | 2 (12.5) | 7 (43.8) |

### Part 2: Pre-meeting survey questions and results

1. How would you rate the priority level for additional/serial CCL14 testing in the following situations? (Single answer for each situation: 1. Not a priority, 2. Low priority, 3. Medium priority, 4. High priority, 5. Essential)
   1. A prior CCL14 value indicating highest risk for persistent severe AKI(e.g., > 13 ng/mL)
   2. A prior CCL14 value indicating increased risk for persistent severe AKI (e.g., > 1.30 ng/mL and ≤ 13 ng/mL )
   3. A prior low CCL14 value indicating lowest risk of developing persistent severe AKI (e.g., ≤ 1.30 ng/mL)
   4. Clinical instability
   5. Clinical improvement

Results of question 1 are provided in **Figure 4**. **Table 11** presents an overview of the ranking of these results.

**Figure 4.** Level of priority for additional/serial CCL14 testing

**Table 12.** Ranking of the scenarions according to the level of interest to know CCL14 values

| Circumstances to Reassess a CCL14 Biomarker Test | Points  (Max 64) |
| --- | --- |
| A prior CCL14 value indicating increased risk for persistent severe AKI (e.g., > 1.30 ng/mL and ≤ 13 ng/mL) | 45 |
| Clinical instability | 35 |
| A prior low CCL14 value indicating lowest risk of developing persistent severe AKI (e.g., ≤ 1.30 ng/mL) | 25 |
| A prior CCL14 value indicating highest risk for persistent severe AKI (e.g., > 13 ng/mL) | 23 |
| Clinical improvement | 13 |
| The points assigned per circumstance were: Not a Priority = 0 points, Low Priority = 1 point, Medium Priority = 2 points, High Priority = 3 points, Essential = 4 points | |

### Part 2: Voting statements and results

**Table 13.** Level of agreement consensus voting workshop 3, Part 2

| Consensus Statement | Response (N=16) | | | | |
| --- | --- | --- | --- | --- | --- |
|  | Disagree  n (%) | Somewhat Disagree  n (%) | Neither Agree nor Disagree  n (%) | Somewhat Agree  n (%) | Agree  n (%) |
| Patient clinical course, including AKI trajectory, new AKI insults, and baseline CCL14 levels, can influence the need to reassess CCL14 biomarker levels. | 0 (0) | 1 (6.3) | 0 (0) | 3 (18.8) | 12 (75) |
| Patients with initial urinary CCL14 levels > 1.30 ng/mL and ≤ 13 ng/mL are potential candidates for re-testing, particularly in scenarios of clinical instability. | 0 (0) | 0 (0) | 0 (0) | 6 (37.5) | 10 (62.5) |
| Candidates for CCL14 re-assessment can be re-tested 24 to 48 hours after initial CCL14 results. | 0 (0) | 0 (0) | 2 (12.5) | 7 (43.8) | 7 (43.8) |

## Workshop 4: How are quantitative CCL14 biomarker results being interpreted?

### Pre-meeting survey questions and results

How helpful would you rate the CCL14 biomarker results for AKI management adjustment according to the following thresholds? (Single answer for each threshold: 1. Definitely not helpful, 2. Likely not helpful, 3. Equally unhelpful/helpful, 4. Likely helpful, 5. Definitely helpful)

1. CCL14 biomarker results ≤ 1.30 ng/mL
2. CCL14 biomarker results > 1.30 ng/mL and ≤ 13 ng/mL
3. CCL14 biomarker results > 13 ng/mL

Results of question 1 are provided in **Figure 4**. **Table 13** presents an overview of the ranking of these results.

**Figure 5.** Helpfulness of CCL14 biomarker results for AKI management adjustment

**Table 14.** Ranking of the Helpfulness of the CCL14 Biomarker Results for AKI Management Adjustment

| CCL14 Biomarker Result | Points  (Max 64) |
| --- | --- |
| CCL14 biomarker results > 13 ng/mL | 53 |
| CCL14 biomarker results ≤ 1.30 ng/mL | 46 |
| CCL14 biomarker results > 1.30 ng/mL and ≤ 13 ng/mL | 35 |
| The points assigned per CCL14 cut-off value were: Definitely Not Helpful = 0 points, Likely Not Helpful = 1 point, Equally Unhelpful/Helpful = 2 points, Likely Helpful = 3 points, Definitely Helpful = 4 points | |

### Voting statements and results

**Table 15.** Level of agreement consensus voting workshop 4

| Consensus Statement | Response (N=16) | | | | |
| --- | --- | --- | --- | --- | --- |
|  | Disagree  n (%) | Somewhat Disagree  n (%) | Neither Agree nor Disagree  n (%) | Somewhat Agree  n (%) | Agree  n (%) |
| Values of urinary CCL14 levels above the high cut-off can be or are likely to be useful for AKI management adjustment. | 1 (6.3) | 0 (0) | 1 (6.3) | 7 (43.8) | 7 (43.8) |
| Values of urinary CCL14 levels above the low cut-off can be or are likely to be useful for AKI management adjustment. | 1 (6.3) | 2 (12.5) | 2 (12.5) | 7 (43.8) | 4 (25) |
| Values of urinary CCL14 levels below the low cut-off can be or are likely to be useful for AKI management adjustment. | 1 (6.3) | 0 (0) | 2 (12.5) | 7 (43.8) | 6 (37.5) |
| Values of urinary CCL14 levels ≤ 1.30 ng/mL help identify patients at the lowest risk of developing PS-AKI and may help de-escalation or maintain AKI management strategies and/or care processes. | 0 (0) | 1 (6.3) | 1 (6.3) | 7 (43.8) | 7 (43.8) |
| Values of urinary CCL14 levels > 1.30 ng/mL and ≤ 13 ng/mL help identify patients at increased risk of developing PS-AKI and may help maintain or escalate AKI management strategies and/or care processes. | 0 (0) | 2 (12.5) | 0 (0) | 10 (62.5) | 4 (25) |
| Values of urinary CCL14 levels > 13 ng/mL help identify patients at the highest risk of developing PS-AKI who could be candidates for increased prioritization of AKI management and care processes. | 0 (0) | (0) | 0 (0) | 4 (25) | 12 (75) |
| Values of urinary CCL14 levels > 13 ng/mL can be or are likely to be useful to escalate AKI management or escalate the level of care and allocated resources. | 0 (0) | 0 (0) | 2 (12.5) | 3 (18.8) | 11 (68.8) |

## Workshop 5: What actions are taken based on CCL14 biomarker results?

### Voting statements and results

**Table 16.** Ranking of the Treatment Areas where CCL14 Results might be Helpful in Informing on Decisions

| Treatment Area | Points  (Max 64) |
| --- | --- |
| Discussions on renal replacement therapy (RRT) initiation | 55 |
| Fluid management (volume) | 44 |
| Diuretic management | 43 |
| Discussions with family on patient prognosis | 43 |
| Hemodynamic management/monitoring | 42 |
| Exposure to nephrotoxins | 42 |
| Drug dosing | 40 |
| Follow-up medical planning after discharge (outpatient care) | 40 |
| Enhance diagnostic work-up (e.g., labs, images) | 38 |
| Strict urine output monitoring (e.g., foley catheter, continuous urinary output monitoring) | 38 |
| Nephrology consultation | 31 |
| Nutritional management | 30 |
| Fluid management (type of solution) | 25 |
| The points assigned per treatment area were: Definitely Not Helpful = 0 points, Likely Not Helpful = 1 point, Equally Unhelpful/Helpful = 2 points, Likely Helpful = 3 points, Definitely Helpful = 4 points | |

**Table 17.** Level of Concern about Modifying Several Areas of the Treatment Plan

|  | Points  (Max 64) | | |
| --- | --- | --- | --- |
| Treatment Plan Area | CCL14  ≤ 1.30 ng/mL  Median (IQR) | CCL14  > 1.30 ng/mL  and ≤ 13 ng/mL  Median (IQR) | CCL14  > 13 ng/mL  Median (IQR) |
| Discussions on renal replacement therapy initiation | 1 (0; 1.25) | 3 (2; 3) | 3 (3; 4) |
| Discussions with family on patient prognosis | 2 (0; 2.25) | 2.5 (1.75; 3) | 3 (2.5; 3) |
| Diuretic management | 1 (0; 2.25) | 2 (2; 3) | 3 (2; 3.25) |
| Drug dosing | 1 (0; 3) | 2 (1.75; 3) | 3 (1.75; 3.25) |
| Enhance diagnostic work-up (e.g. labs, images) | 1 (0; 2) | 2.5 (2; 3) | 3 (2.75; 3.25) |
| Exposure to nephrotoxins | 1.5 (0; 2.25) | 3 (2; 3) | 3 (2.75; 4) |
| Fluid management (type of solution) | 0.5 (0; 2.25) | 1.5 (1; 2.25) | 3 (1; 3.25) |
| Fluid management (volume) | 1 (0; 2.25) | 2 (2; 3) | 3 (3; 4) |
| Follow-up medical planning after discharge (outpatient care) | 1 (0; 2) | 2.5 (1; 3) | 3 (1; 3) |
| Hemodynamic management/monitoring | 1 (0; 2.25) | 2.5 (2; 3) | 3 (2.75; 4) |
| Nephrology consultation | 0 (0; 1) | 2 (1; 3) | 2.5 (1; 3) |
| Nutritional management | 0.5 (0; 2) | 2 (1; 2.25) | 2 (1; 3) |
| Strict urine output monitoring (e.g. foley catheter, continuous urinary output monitoring) | 0.5 (0; 2.25) | 3 (2; 3) | 3 (2.75; 4) |
| The points assigned per treatment area were: Not At All Concerned = 0 points, Slightly Concerned = 1 point, Moderately Concerned = 2 points, Very Concerned = 3 points, Extremely Concerned = 4 points. | | | |

**Post-Hoc Analyses**

A post-hoc analysis was performed on the difference between the CCL14 cut-off values relating to the level of concern on modifying the treatment plan. The mean and standard deviation of total score per CCL14 group are as follows: ≤1.3 ng/mL (Low): 18.8 ± 2.89; >1.3 ng/mL to ≤13 ng/mL (Medium): 36.4 ± 4.44; and >13 ng/mL (High): 41.7 ± 4.99. Similar, the mean and standard deviation of level of concern assigned on Likert Scale to each cut-off level are as follows: ≤1.3 ng/mL (Low): 1.2 ± 1.23; >1.3 ng/mL to ≤13 ng/mL (Medium): 2.3 ± 1.04; and >13 ng/mL (High): 2.61 ± 1.29.
